# Increased Ca^2+^ content of the sarcoplasmic reticulum provides arrhythmogenic trigger source in swimming-induced rat athlete’s heart model

**DOI:** 10.1038/s41598-020-76496-2

**Published:** 2020-11-11

**Authors:** Péter Gazdag, Kinga Oravecz, Károly Acsai, Vivien Demeter-Haludka, Balázs Ördög, Jozefina Szlovák, Zsófia Kohajda, Alexandra Polyák, Bálint András Barta, Attila Oláh, Tamás Radovits, Béla Merkely, Julius Gy. Papp, István Baczkó, András Varró, Norbert Nagy, János Prorok

**Affiliations:** 1 Department of Pharmacology and Pharmacotherapy, Faculty of Medicine, University of Szeged, Dóm tér 12, P.O. Box 427, Szeged, 6720 Hungary; 2 MTA-SZTE Research Group of Cardiovascular Pharmacology, Hungarian Academy of Sciences, Szeged, Hungary; 3 2nd Department of Internal Medicine and Cardiology Centre, Faculty of Medicine, University of Szeged, Szeged, Hungary; 4 Experimental Research Laboratory, Heart and Vascular Center, Semmelweis University, Budapest, Hungary; 5 Department of Pharmacology and Pharmacotherapy, Interdisciplinary Excellence Centre, University of Szeged, Szeged, Hungary

**Keywords:** Cardiology, Medical research, Risk factors

## Abstract

Sudden cardiac death among top athletes is very rare, however, it is 2–4 times more frequent than in the age-matched control population. In the present study, the electrophysiological consequences of long-term exercise training were investigated on Ca^2+^ homeostasis and ventricular repolarization, together with the underlying alterations of ion channel expression, in a rat athlete's heart model. 12-week swimming exercise-trained and control Wistar rats were used. Electrophysiological data were obtained by using ECG, patch clamp and fluorescent optical measurements. Protein and mRNA levels were determined by the Western immunoblot and qRT-PCR techniques. Animals in the trained group exhibited significantly lower resting heart rate, higher incidence of extrasystoles and spontaneous Ca^2+^ release events. The Ca^2+^ content of the sarcoplasmic reticulum (SR) and the Ca^2+^ transient amplitude were significantly larger in the trained group. Intensive physical training is associated with elevated SR Ca^2+^ content, which could be an important part of physiological cardiac adaptation mechanism to training. However, it may also sensitize the heart for the development of spontaneous Ca^2+^ release and extrasystoles. Training-associated remodeling may promote elevated incidence of life threatening arrhythmias in top athletes.

## Introduction

Many clinical and epidemiological studies provided evidence that moderate physical exercise markedly improves cardiovascular function, decreases mortality and prevents sudden cardiac death^1–3^. In contrast, highly increased physical exercise performed regularly by competitive top athletes causes structural remodeling of the left ventricle, including cardiac hypertrophy (structural remodeling)^4,5^, alterations in the ion channel densities, possibly causing electrical instability (electrical remodeling), bradycardia^6^ and arrhythmias. These alterations accompanied with a preserved ejection fraction have been classically termed as physiology of the “athlete’s heart”^7^. Although sudden cardiac death in top athletes is very rare^8^, it is 2–4 times more frequent than in the age-matched control population^9^ and is mostly attributed to ventricular fibrillation. Therefore, long-term high intensity endurance exercise may produce increased arrhythmia sensitivity associated with sudden cardiac death^2,10^. One of the major suspected cause of sudden cardiac death in top athletes is hypertrophic cardiomyopathy which generates high electrical instability in ventricular tissues^11^.

Several studies investigated the electrophysiological consequences of intensive exercise training and provided evidence that training can distort the normal transmural repolarization heterogeneity primarily by inducing changes in I_to_ density and ameliorating Ca^2+^ handling abnormalities^12^. Furthermore, repolarization attenuation was also reported^13,14^. In spite of these data, the electrophysiological consequences of cardiac adaptation during intensive exercise training are still controversial. We hypothesized that sudden cardiac death in top athletes could be the consequence of the parallel existence of a trigger mechanism, such as delayed afterdepolarizations, and repolarization inhomogeneity, representing an enhanced arrhythmogenic substrate^15^.

Our aim was therefore to investigate, in a rat athlete’s heart model, the role of selected components of exercise-induced cardiac adaptation in the increased arrhythmia propensity.

## Materials and methods

### Animals

All experimental procedures were reviewed and approved by Ethical Committee of Hungary for Animal Experimentation in accordance with the ‘‘Principles of Laboratory Animal Care’’ defined by the National Society for Medical Research (permission number: PEI/001/2374-4/2015) and the Guide for the Care and Use of Laboratory Animals, provided by the Institute of Laboratory Animal Resources and published by the National Institute of Health (NIH Publication No. 85-23, revised 1996.); and to the EU Directive 2010/63/EU guidelines. All animals received human care.

8-week-old Wistar male rats (Toxi-Coop, Dunakeszi, Hungary) (n = 36, m = 240-280 g) were housed in standard rat cages at a constant room temperature (22 ± 2 °C) with 12/12-h light/dark cycles. Rats received standard laboratory diet and water ad libitum. The body weight of animals was controlled regularly (three times a week).

### Exercise training protocol

Following acclimatization the rats were randomly divided into control (n = 18) and trained groups (n = 18). Animals of the trained group underwent a 12-week-long swimming training protocol to induce physiological myocardial hypertrophy as described previously^16^. In brief, swimming training was performed 5 days/week in a divided container filled with tap water (45 cm deep) maintained at 30–32 °C. For adaptation, the duration of swimming was increased by 15 min every second training day from a baseline of 15 min on the first day, until obtaining the maximal 200 min/day. During this 12-week-long period, control animals were accommodated to water 5 min/day, 5 days/week to reduce the possible differences caused by the stress of water contact.

### Echocardiography

At the completion of the swimming training program, LV morphological alterations in control (n = 18) and trained (n = 18) rats were observed by echocardiography as described before^17^, except that rats were anesthetized with isoflurane (5% induction dose, 1–2% maintenance dose). Animals were placed on controlled heating pads, and the core temperature was maintained at 37 °C. After shaving the anterior chest, transthoracic echocardiography was performed in the supine position using a 13 MHz linear transducer (12L-RS, GE Healthcare, Horten, Norway), connected to an echocardiography system (Vivid i, GE Healthcare). Standard two-dimensional and M-mode long- and short axis (at mid-papillary level) images were acquired. Recordings were analyzed off-line using a dedicated software (EchoPac, GE Healthcare). We calculated heart rate (HR) on images recorded by M-mode. On two-dimensional recordings of the short-axis at the mid-papillary level, LV anterior (AWT) and posterior (PWT) wall thickness in diastole (index: d) and systole (index: s) as well as LV end-diastolic (LVEDD) and end-systolic diameter (LVESD) were measured. End-systole was defined as the time point of minimal LV dimensions, while end-diastole as the time point of maximal dimensions. All values were averaged over three consecutive cycles^17^.

Fractional shortening (FS) was determined from the measurements of LV chamber diameters: FS = [(LVEDD-LVESD)/LVEDD] × 100. LV mass was calculated according to the following formula: LVmass = [(LVEDD + AWTd + PWTd)^3 ^− LVEDD^3^] × 1.04 × 0.8 + 0.14. To calculate LV mass index, we normalized the LV mass values to the tibial length (TL) of the animal^17^.

### Morphometric assessment

Standard morphometric measurements were obtained including body weight and post-mortem heart weight, as well as tibial length. All animals were weighed before termination. At the end of Langendorff isolated heart measurements, the dry heart weights were measured (n = 12/group). Routinely prepared tibia length was measured after termination. For morphometric analysis, were using a conventional analytical balance and a ruler.

### Isolated heart experiments

After 12-week-long swimming training 20-week-old male Wistar rats were used (12 control and 12 trained). ECG and left ventricular pressure of isolated hearts were measured in Langendorff-perfused hearts as described before^18^. Animals were anaesthetized with Na-pentobarbital (300 mg/kg, i.p.), and were injected with heparin sodium (300 IU) into the portal vein. Hearts were rapidly excised, mounted via the aorta on a Langendorff apparatus and retrogradely perfused with warm (37 °C) modified Krebs–Henseleit bicarbonate buffer (KHB) at a constant pressure (80 Hgmm). The KHB solution contained (in mmol/L): NaHCO_3_ 25; KCl 4.3; NaCl 118.5; MgSO_4_ 1.2; KH_2_PO_4_ 1.2; glucose 10; CaCl_2_ 1.8, having a pH of 7.4 ± 0.05 when gassed with 95% O_2_ + 5% CO_2_. The left ventricular pressure (LVP) was measured by a water-filled latex balloon which was inserted into the left ventricular cavity and inflated to obtain a control state end-diastolic pressure (LVEDP) of 4–8 mmHg^18^. The constant column pressure was provided by a pump (Masterflex) continuously changing the KHB.

The electrical activity as electrocardiogram (ECG) detected by the three lead self-made electrodes and signal amplifier (Experimetria, Hungary). The LVP and the ECG were simultaneously recorded using the WinWCP software (V4.9.1. Whole Cell Electrophysiology Analysis Program, John Dempster, University of Strathclyde, UK). Ventricular extrasystoles were induced by hypokalemic (2.7 mM K^+^) KHB solution.

### Measurement of ionic currents

Rat ventricular cardiomyocytes were isolated as described in our previous study^19^. The L-type Ca^2+^ current, K^+^ currents, Ca^2+^ transient measurements were also described earlier^20^. The estimation of sarcoplasmic reticulum Ca^2+^ content by caffeine method was applied as previously described^21^.

### Determination of phospho-PKA C, phospho-phospholamban and SERCA2 by western blot

The pan and phosphorylated forms of PKA C, phospholamban (PLN) and SERCA2 were measured in myocardial tissue samples taken from the left ventricle (n = 6/group). Fresh tissue samples were immediately frozen in liquid nitrogen and stored at -80 °C. 30 µg (PKA C, pPKA C), 50 µg (PLN, pPLN) and 20 µg (SERCA2) total protein extracts were resolved using 10% (PKA C, pPKA C), 15% (PLN, pPLN) and 8% (SERCA2) sodium dodecyl sulphate–polyacrylamide gel electrophoresis and transferred onto polyvinylidene fluoride membranes. After blocking in 5% milk-TBS-T, the membranes were immunolabeled with the respective primary antibodies provided by the Calcium Ion Regulation Antibody Sampler Kit (Cell Signalling Technology; Danvers, MA, USA; overnight, at 4 °C; dilutions: anti-PKA C, anti-pPKA C (-α, -β, and -γ when phosphorylated at Thr197): 1:7000, anti-PLN, anti-pPLN (when phosphorylated at Ser16/Thr17): 1:2500, anti-SERCA2: 1:7000). Horseradish peroxidase-conjugated goat anti-rabbit IgG (Southern Biotech, Birmingham, AL, USA; 1 h, RT; 1:8000) was used as a secondary antibody. The membranes were developed with an ECL kit (Advansta; San Jose, CA, USA) and exposed to X-ray film. Equal protein loading was verified by coomassie blue staining, and normalized to total protein. Equal protein loading was verified by coomassie blue staining. Integrated optical density values (sum of each band corrected to the background) was assessed using Image J (FIJI; NIH, Bethesda, MD, USA).

### Gene expression analysis by qRT-PCR

All mRNA analyses were carried out as described previously^22^. Fresh left ventricular tissue samples (n = 6/group) were excised and snap-frozen in liquid nitrogen and stored at − 80 °C. Myocardial samples were homogenized in a lysis buffer (RLT buffer; Qiagen, Hilden, Germany), total RNA was isolated from the tissue using the RNeasy Fibrous Tissue Mini Kit (Qiagen) according to the manufacturer’s instructions and quantified by measuring optical density at 260 nm. 1 µg total RNA was used for reverse transcription [QuantiTect Reverse Transcription Kit (Qiagen)]. Quantitative real-time PCR was performed with the StepOnePlus Real-Time PCR System (Applied Biosystems, Foster City, CA, USA) in triplicates of each sample, in the total volume of 10 µl in each well containing cDNA, TaqMan Universal PCR MasterMix and a TaqMan Gene Expression Assay for the following genes: alpha-1 subunit of a voltage-dependent calcium channel (Cacna1c, assay ID: Rn00709287_m1), alpha-2 and delta subunits of the voltage-dependent calcium channel complex (Cacna2d1, Rn01442580_m1), beta-2 subunit of the voltage-dependent calcium channel complex (CACNB2, Rn00587789_m1), ryanodine receptor 2 (Ryr2, Rn01470303_m1), calsequestrin 2 (CASQ2, Rn00567508_m1), Na^+^/Ca^2+^exchanger (NCX) SLC8A1, Rn04338914_m1), sarco/endoplasmic reticulum Ca^2+^-ATPase (SERCA2) (Atp2a2, Rn00568762_m1) and phospholamban (PLN, Rn01434045_m1) purchased from Applied Biosystems. Data were normalized to glyceraldehyde-3-phosphate dehydrogenase (GAPDH; assay ID: Rn01775763_g1) and expression levels were calculated using the CT comparative method (2^-ΔCT^). All results are expressed as values normalized to the average values of the control group.

### Statistical analysis

All data are presented as mean ± SEM. To compare post-mortem, morphological, hemodynamic parameters and ionic currents of control and trained animals, unpaired Student’s t-test was used. *p* ≤ 0.05 was considered to be statistically significant.

## Results

### Echocardiographic results

**Figure 1:**
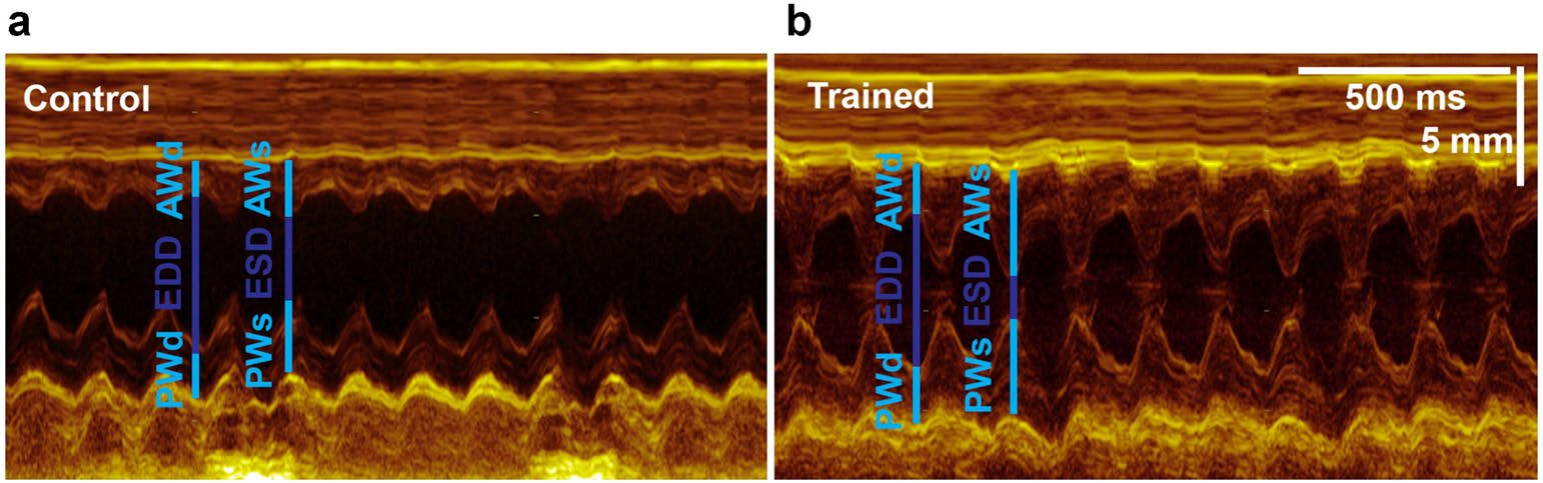
Representative left ventricular (LV) M-mode recordings from one control and one trained animal. Exercise training was associated with increased wall thickness values and markedly decreased LV end-systolic diameter.

The echocardiographic results are shown in Table 1. The resting heart rate (HR) was significantly decreased in the trained rats, compared to control animals. Echocardiography also revealed significant myocardial hypertrophy, with increased left ventricular (LV) anterior and posterior wall thickness both in systole and diastole, as well as LV mass index. Unchanged LV end-diastolic and decreased end-systolic dimensions resulted in a considerably higher fractional shortening in trained rats, suggesting increased systolic performance (Fig. 1).

**Table 1:** Echocardiographic and morphometric data.

	Control (n = 18)	Trained (n = 18)	*p*
HR (beat/s)	371 ± 6	314 ± 8	< 0.05
LVAWTd (mm)	2.07 ± 0.04	2.31 ± 0.05	< 0.05
LVAWTs (mm)	3.16 ± 0.07	3.67 ± 0.09	< 0.05
LVPWTd (mm)	1.98 ± 0.06	2.24 ± 0.06	< 0.05
LVPWTs (mm)	2.82 ± 0.09	3.23 ± 0.09	< 0.05
LVEDD (mm)	7.66 ± 0.10	7.53 ± 0.13	0.415
LVESD (mm)	4.62 ± 0.10	4.17 ± 0.18	< 0.05
FS (%)	38.9 ± 1.4	45.4 ± 1.8	< 0.05
LV mass (g)	1.19 ± 0.04	1.41 ± 0.05	< 0.05
LV mass index (g/kg)	2.38 ± 0.07	3.21 ± 0.10	< 0.05

	Control (n = 12)	Trained (n = 12)	*p*
Tibial length (mm)	45.6 ± 0.8	45.1 ± 0.7	0.62
Body weight (g)	501.12 ± 12.2	428.85 ± 5.86	< 0.05
Heart weight (g)	1.91 ± 0.08	2.18 ± 0.08	< 0.05
Heart weight index (g/kg)	3.83 ± 0.10	5.1 ± 0.20	< 0.05
Ventricular weight (g)	1.41 ± 0.06	1.63 ± 0.05	< 0.05
Ventricular weight index (g/kg)	2.48 ± 0.10	3.81 ± 0.12	< 0.05

### Morphometric results and Langendorff-perfused experiments

The morphometric data of training induced cardiac hypertrophy, measured at the end of the training program, are shown in Table 1. The unchanged lengths of tibia verified the identical age between control and trained animals. The sedentary rats had significantly larger body weight, whereas physical dimensions of the heart including total weight, weight index, ventricles weight and index were significantly increased in the trained rats.

**Figure 2:**
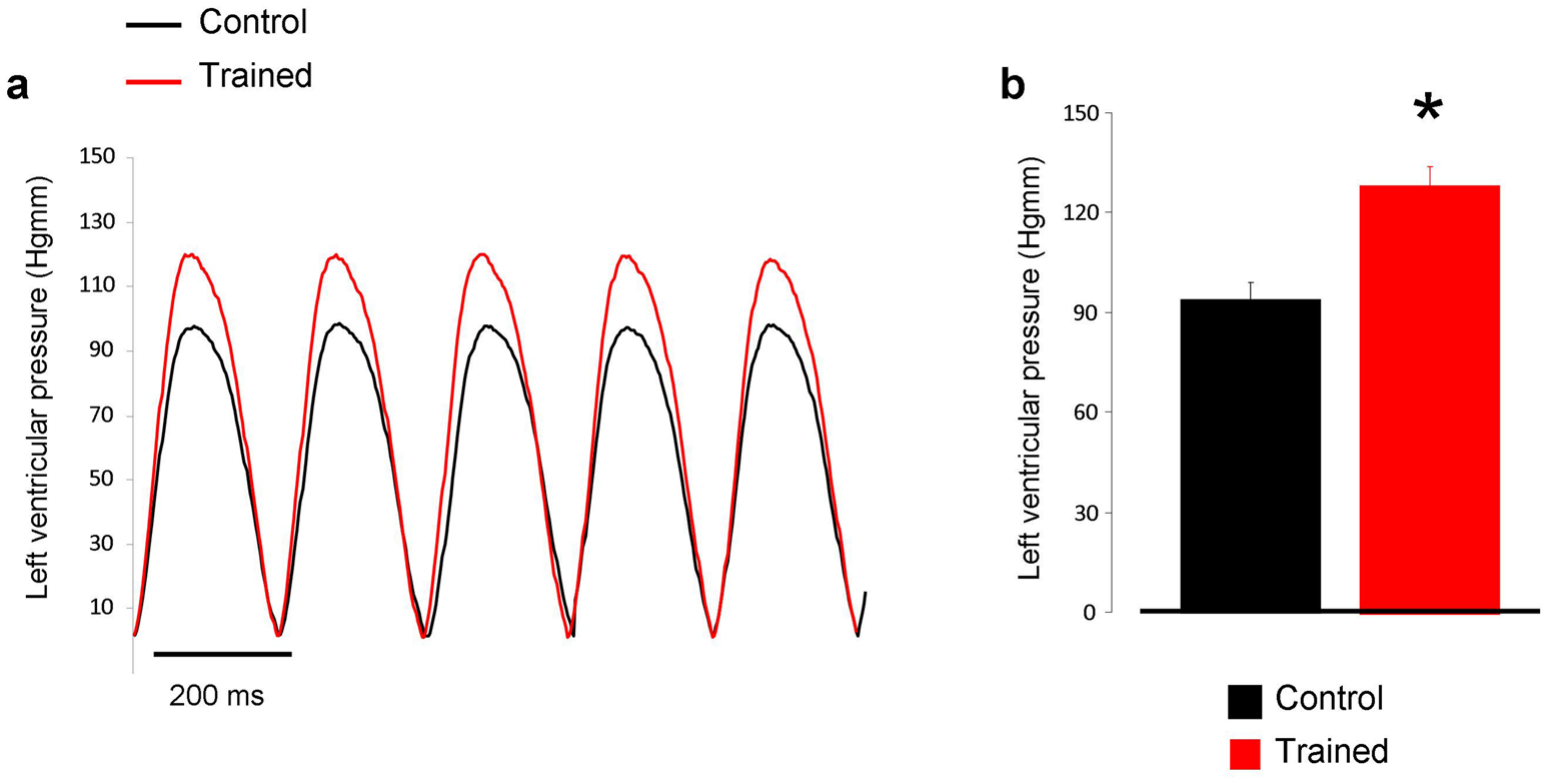
Panel (**a**) demonstrates representative left ventricular developed pressure curves in control (black trace) and in exercised rats (red trace) during Langendorff-perfused measurements. As bar graphs in panel (**b**) illustrate the left ventricular pressure was significantly higher in the case of trained group (red column).

**Figure 3:**
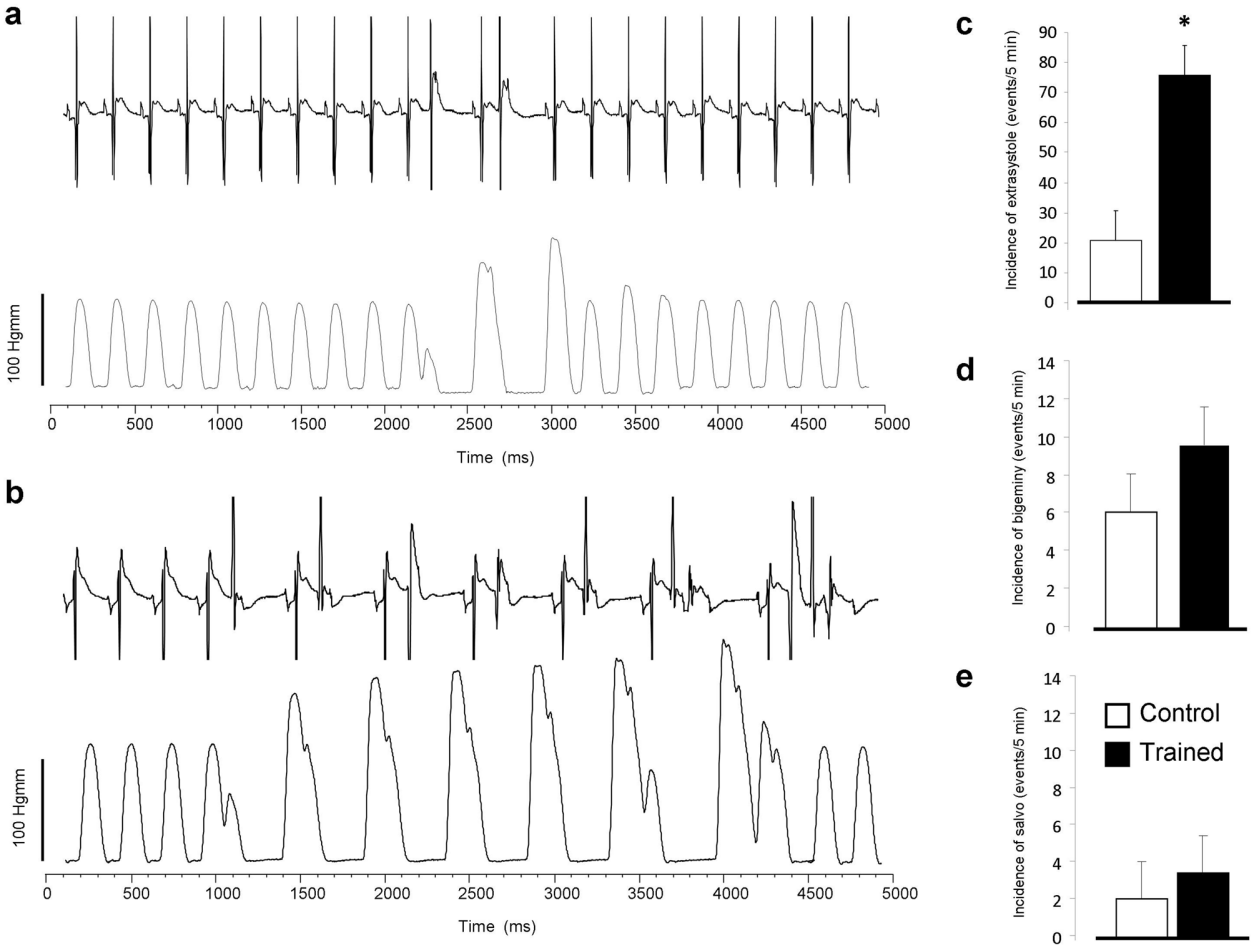
Arrhythmia incidence between trained and control groups measured by parallel registration of ECG and left ventricular pressure during Langendorff-perfusion. A representative section of ECG and pressure from control group indicate few extrasystoles in panel (**a**). In the trained group (panel **b**) the number of extrasystoles significantly increased. Panels (**c**–**e**) compares the extrasystole, bigeminy and salvo incidence between control and trained group, respectively.

The obtained results of *ex-vivo* Langendorff experiments are shown in Table 2. ECG recordings showed significantly increased long-term R-R variability in the trained group compared to control hearts, while R-R intervals remained unchanged between the two groups. Similarly, QT intervals were not different, while the long-term QT variability was decreased in trained rats. In line with the echocardiographic data, the LV end-systolic pressure was found to be larger in trained animals (Fig. 2a,b). The arrhythmia analysis revealed that the trained group exerted significantly more ventricular extra beats (21 ± 4 vs 75 ± 21 extra beats, n = 12, *p* < 0.05). There were no significant difference between groups regarding bigeminy (6 ± 2 vs 10 ± 2 extra beats, n = 12) or salvos (2 ± 1 vs 3 ± 1 salvos, n = 12) (Fig. 3).

**Table 2:** ECG and left ventricular pressure parameters measured from isolated, Langendorff perfused rat hearts.

	Control (n = 12)	Trained (n = 12)	*p*
RR (ms)	210.8 ± 5.76	214.17 ± 5.36	0.670
RRSTV (ms)	0.77 ± 0.13	1.25 ± 0.36	0.21
RRLTV (ms)	0.65 ± 0.06	1.57 ± 0.51	< 0.05
QT (ms)	87.24 ± 7.46	85.43 ± 4.41	0.839
QTSTV (ms)	0.310 ± 0.03	0.258 ± 0.06	0.44
QTLTV (ms)	0.506 ± 0.03	0.363 ± 0.05	< 0.05
LVESP (mmHg)	108.24 ± 6.49	133.56 ± 6.53	< 0.05
LVEDP (mmHg)	4.69 ± 0.93	4.58 ± 0.44	0.924
LVDP (mmHg)	103.55 ± 6.35	128.98 ± 6.19	< 0.05

**Figure 4:**
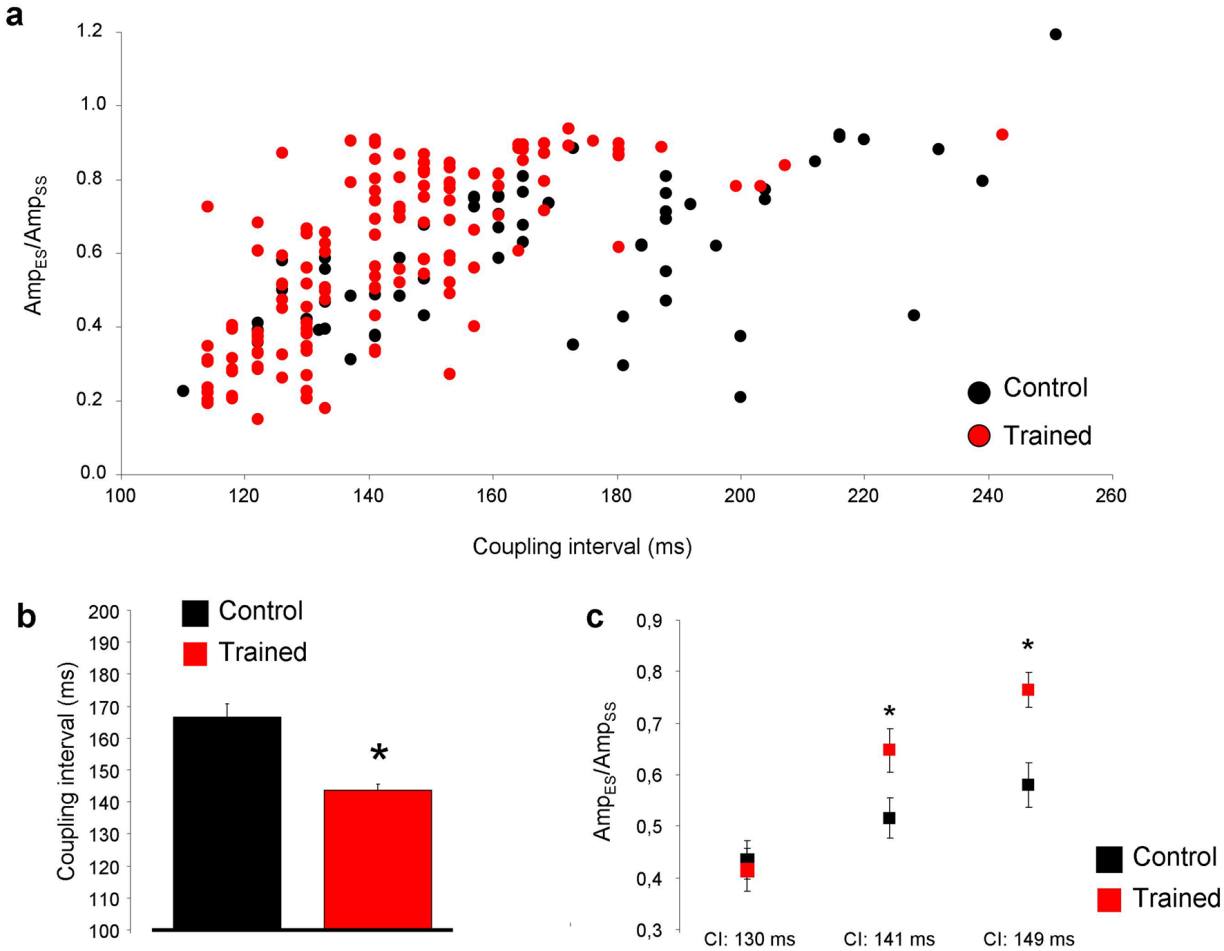
Analysis of premature beats of Langendorff-perfused hearts. (**a**,**b**) show that trained group has shorter coupling interval and larger amplitude of extra beats (**c**) compared to control.

The characteristics of premature beats were further analyzed and the results are demonstrated in Fig. 4. The extra beats were analyzed for a 5-min-long section in all experiments. Only the clearly separated single extra beats were involved in the data analysis, bigeminy and salvo were excluded. Panel a represents the distribution of single extra beats/steady-state beats amplitude ratio in the function of the corresponding coupling interval. The coupling intervals were determined as the time between the initiation of the extra beat and the initiation of the upstroke of the previous steady-state beat. The trained animals (red dots in panel a, and red column in panel b) exerted significantly shorter coupling intervals compared to control (143.7 ± 1.86 ms vs 166.5 ± 4.12 ms; panel b; *p* < 0.05, n = 135 and 63 respectively, both from 12 animals). The amplitude ratio of premature beats/steady-state beats was compared at 3 discrete coupling intervals, where we could gather sufficient number of data (130, 141 and 149 ms). Since the control group exerted only a few numbers of extra beats at these intervals, we extended the analysis of control group to 10 min. As illustrated in panel c, the ratio of amplitudes were slightly larger in trained animals in 141 ms (0.64 ± 0.04 vs 0.51 ± 0.03, *p* < 0.05, n = 18 and 12 respectively, both from 12 animals) and 149 ms (0.76 ± 0.03 vs 0.58 ± 0.04, *p* < 0.05, n = 12 and 13 respectively both from 12 animals) compared to control.

### Measurement of spontaneous Ca^2+^ releases

**Figure 5:**
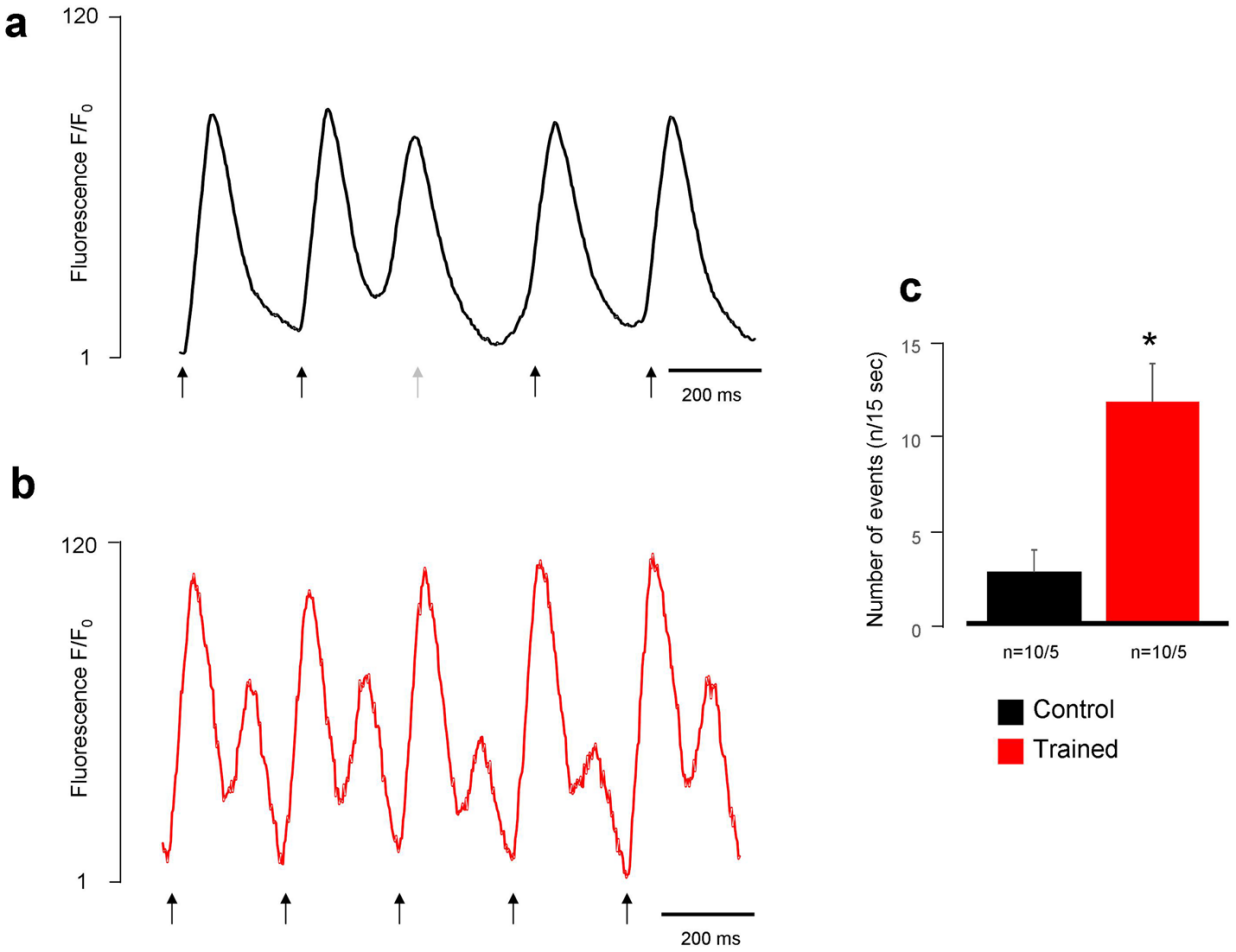
Comparison of spontaneous Ca^2+^ releases under 4 Hz stimulation frequency between control (**a**) and trained (**b**) rats. The black arrows indicate the electric stimuli, the grey arrow marks an ineffective stimulus. We found larger number of spontaneous Ca^2+^ releases in the trained group, compared to control (**c**).

Spontaneous Ca^2+^ release was measured, in single cardiomyocytes field-stimulated at 4 Hz for 15 s. Although we observed spontaneous Ca^2+^ release episodes in both groups, the number of spontaneous events was significantly larger in the trained group (11.7 ± 3.9 events/15 s vs 2.7 ± 1.2 events/15 s, n = 10/5 and 10/5 respectively, *p* < 0.05, Fig. 5).

### The I_Ca,L_, SR Ca^2+^ content and Ca^2+^ transient measurements

**Figure 6:**
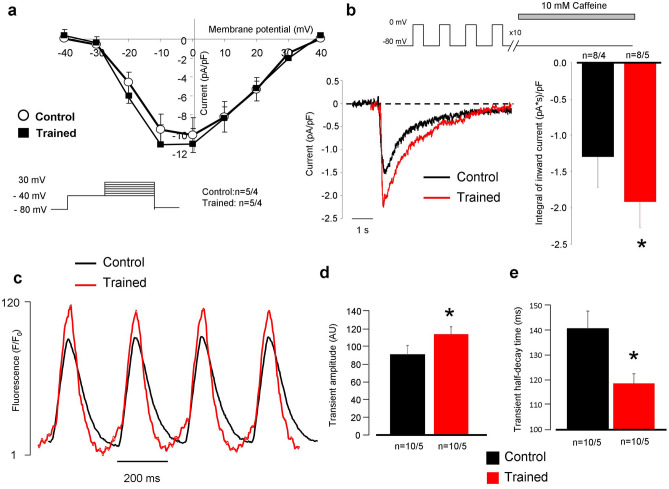
Assessment of Ca^2+^ handling on isolated cells. Panel (**a**) shows identical current–voltage relationship of L-type Ca^2+^ current between groups. Panel (**b**) illustrates significantly larger inward current as a response of 10 mM caffeine application. Panel (**c**,**d**) reports larger Ca^2+^ transient amplitude in the case of trained rats (red trace) compared to control (black trace). Panel (**e**) indicates faster transient relaxation kinetics in the case of trained animals.

Figure 6a shows the voltage-current relationship of the L-type Ca^2+^ current (I_Ca,L_) in the presence of buffered intracellular solution. 50 ms-long depolarization pulses from a holding potential of − 80 mV to − 40 mV were applied to inactivate the sodium current, followed by voltage steps to 30 mV to elicit Ca^2+^ current. As the superimposed plot and the bar graph (at + 10 mV) show, I_Ca,L_ density was not different in the trained group at all membrane potentials compared to control (n = 5/4 and n = 5/4). Rapid application of 10 mM caffeine (Fig. 6b) at a holding potential of − 80 mV was used to estimate the SR Ca^2+^ content. The caffeine flush was preceded by 10 consecutive conditioning pulses from -80 to 0 mV to reach a steady-state SR Ca^2+^ level. We analyzed the integral of caffeine-induced NCX currents as an indicator of the SR Ca^2+^ content and found that SR Ca^2+^ content was significantly increased in the trained group compared to controls (− 1.84 ± 0.4 (pA*s)/pF vs − 1.25 ± 0.5 (pA*s)/pF n = 8/5 and 8/4, respectively, *p* < 0.05; Fig. 6b). Ca^2+^ transients were measured at 4 Hz pacing frequency to approximate the physiological heart rate of rats (Fig. 6c). We found that the magnitudes of Ca^2+^ transients obtained from the trained group were increased compared to control animals (trained: 114.1 ± 8 AU vs control: 91.1 ± 10 AU, n = 10/5 and 10/5, respectively, *p* < 0.05, Fig. 6d). The half-decay time of the Ca^2+^ transients, measured at 50% of transient decay, was faster in the case of the trained group (118.7 ± 4 ms vs 140.8 ± 5 ms, n = 10, *p* < 0.05, Fig. 6e).

### Repolarizing potassium currents, I_to_ and I_K1_

**Figure 7:**
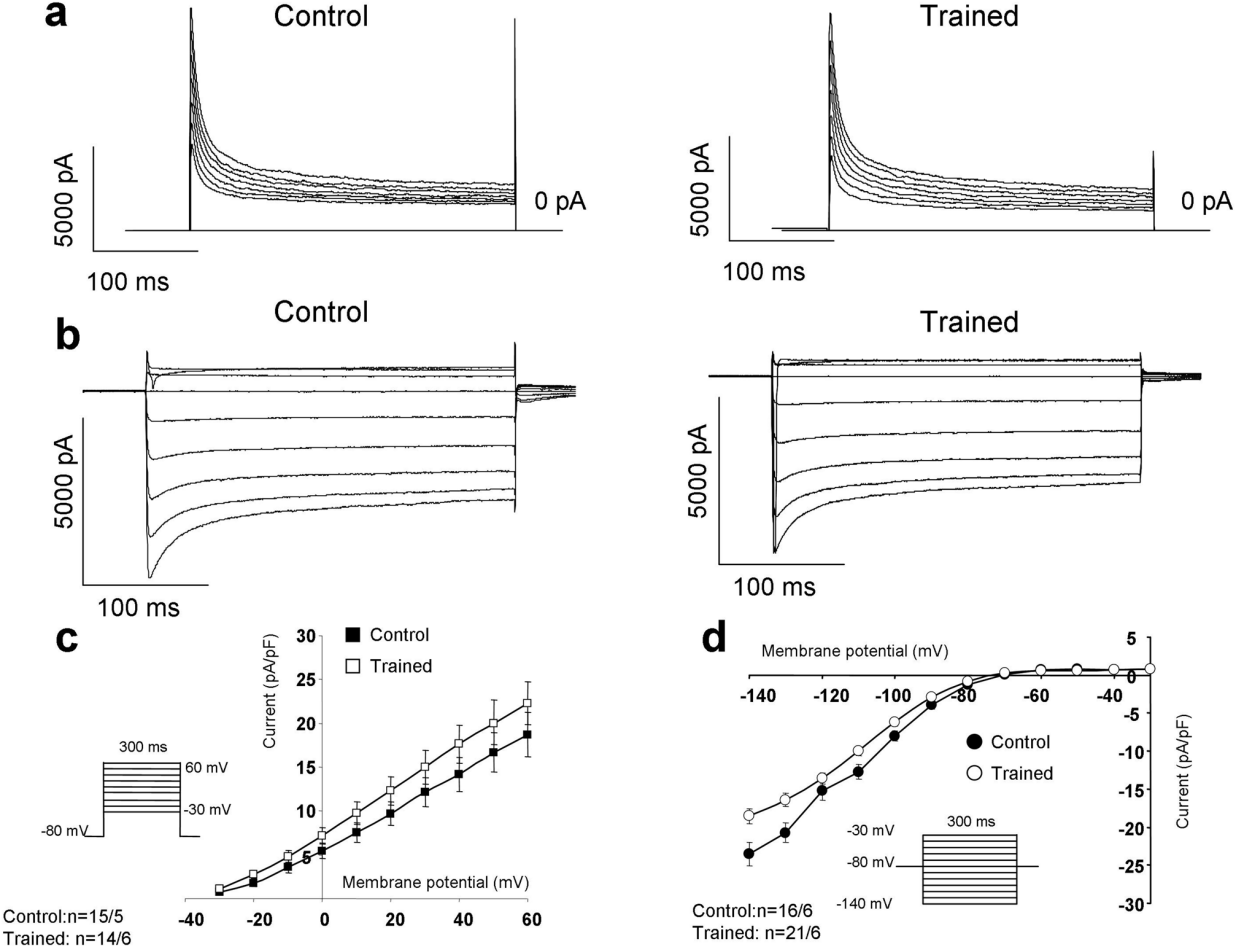
Investigation of the main repolarizing potassium currents on isolated cells. As representative current traces (Panel **a**,**b**) and current–voltage diagrams (panel **c**) illustrate, the currents were found identical between control and trained groups.

The potential remodeling induced changes in the densities of the I_to_ and I_K1_ were examined in the presence of 10 mM EGTA and I_CaL_ inhibition. I_to_ (Fig. 7a,c) was elicited by 300 ms-long voltage steps to 60 mV from a holding potential of -80 mV. As original current traces (Fig. 7a), as well as current–voltage diagram (Fig. 7c) show, the currents were almost identical between groups. I_K1_ was measured by using 300 ms-long depolarizing pulses between − 140 and − 30 mV from a holding potential of -80 mV. Similarly to I_to_, I_K1_ did not differ between the control and trained groups (Fig. 7b,d).

Average cell size, as estimated by the whole cell capacitance obtained from our patch clamp experiments was increased significantly, in cardiomyocytes from trained animals compared to control cells (281 ± 12 pF vs 232 ± 15 pF, n = 20, *p* < 0.05) (Figs. 6a and 7).

### Ion channel gene expression levels

**Figure 8:**
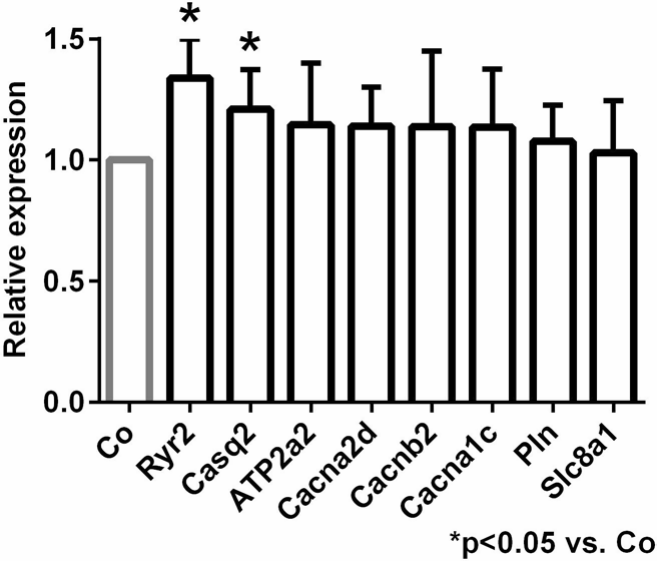
Myocardial gene expression analysis.

The expression levels of genes involved in Ca^2+^ handling were examined by qRT-PCR. We found that the relative mRNA expression of ryanodine receptor 2 and calsequestrin were significantly higher in the trained group compared to control. The mRNA levels of NCX, SERCA2, LTCC genes and PLN were not different (Fig. 8).

### Phosphorylation of PKA C, PLN and SERCA2 protein expression

**Figure 9:**
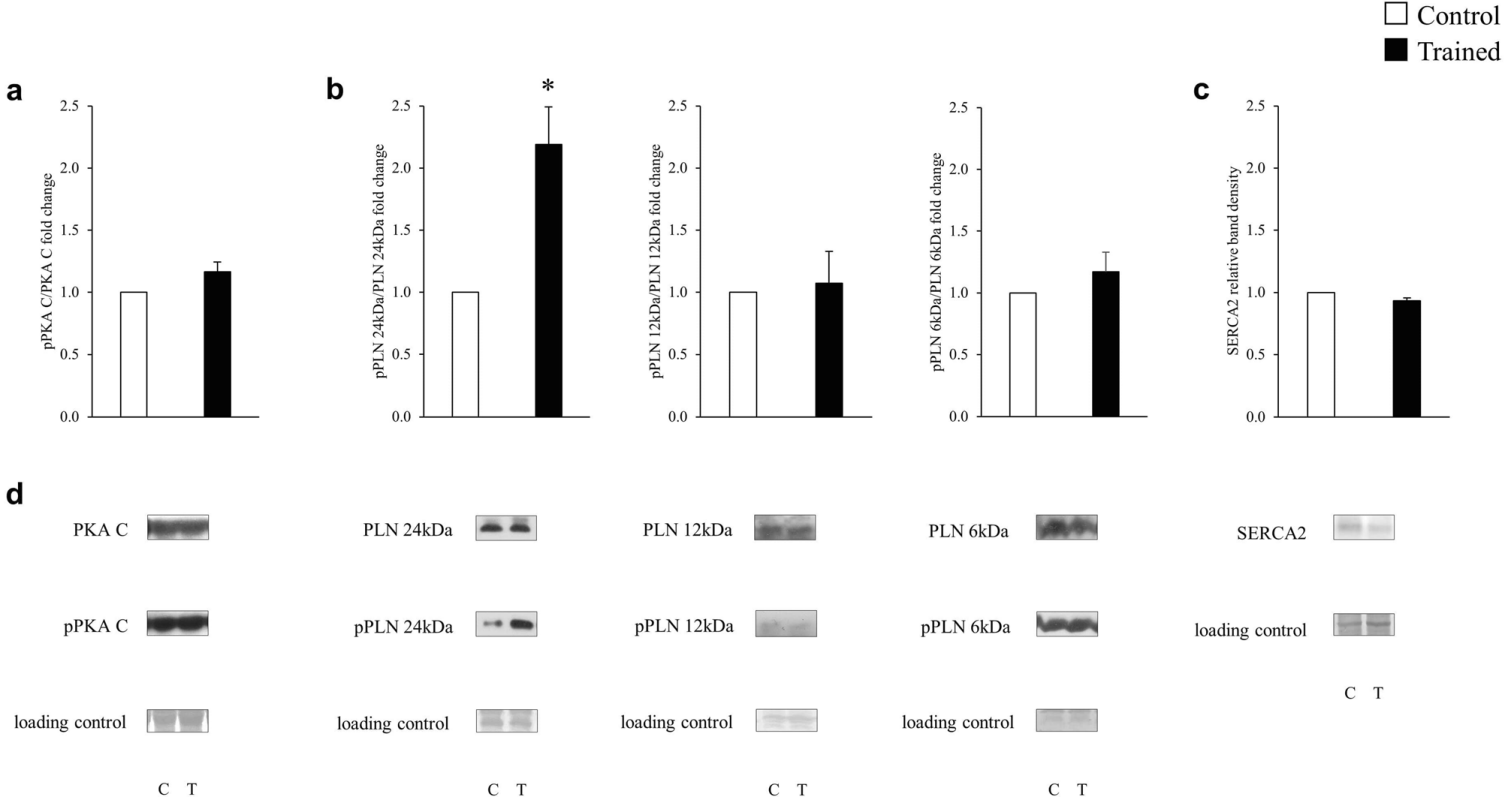
The effect of training on the SERCA phosphorylation pathway. The pan and phosphorylated PKA C (**a**), PLN (**b**) and the SERCA2 (**c**) protein expression.

The pan and phosphorylated forms of key proteins involved in the regulation of Ca^2+^ homeostasis, including PKA C, PLN and SERCA2 protein expression were compared in biopsies from the left ventricles of trained and control rats. Training markedly increased phosphorylation of phospholamban oligomers. There were no significant differences between the groups regarding PKA C phosphorylation, and SERCA expression (Fig. 9).

## Discussion

In this work, we studied the consequences of structural and electrical remodeling induced by intensive exercise training in rats. The most important findings of the study are: (i) ventricular hypertrophy and increased cardiac output in trained rats is associated with enhanced arrhythmogenic trigger activity; (ii) these arrhythmogenic events are predominantly of ventricular origin, suggesting delayed afterdepolarizations via increased SR Ca^2+^ content as an underlying mechanism; (iii) the enhanced SR Ca^2+^ content is maintained by increased expression of the phosphorylated form of phospholamban, and increased level of calsequestrin.

Our results suggest that the physiological adaptation of Ca^2+^ homeostasis underlying the increased cardiac output demand during exercise, provides a potentially harmful arrhythmia source. In our study, neither key repolarization parameters nor the repolarization inhomogeneity^15^ differ significantly in trained compared to control rats, which suggests no change in the arrhythmia substrate in the rat model. However, the increased triggering activity observed in the rat model of the athlete’s heart may be coupled with increased transmural dispersion of repolarization in humans (e.g.: as a possible consequence of certain medications and/or dietary supplements, steroid doping agents, or congenital genetic disorders) and therefore can be accounted for the development of life threatening arrhythmias in top athletes. It should be noted that, in contrast to other mammals including human, neither the rapid nor the slow (I_Ks_) delayed rectifier potassium currents (I_Kr_ and I_Ks_, respectively) operate in the rat. Therefore, these currents could not be studied in our model, warranting further studies using other species (e.g. rabbits, dogs) to investigate possible changes in repolarization potentially enhancing the arrhythmia substrate during endurance training.

### Increased vagal tone may underlie sinus bradycardia

Sinus bradycardia is a hallmark characteristic of the athlete’s heart^3,23^ as it was the case in several animal training models^6,24,25^. Sinus bradycardia is generally considered as the consequence of increased vagal tone^26^, however, D’Souza et al^6^ demonstrated the down-regulation of the pacemaker (funny) current (I_f_) after the training period of running-trained rats. In our experiments, sinus bradycardia was found in the setting of *in-vivo* echocardiographic measurements (Table 1), but not in Langendorff-perfused isolated hearts (Table 2). The apparent discrepancy between our study and that of D’Souza^6^ is may be due to the fundamental differences between the training regime (swimming vs treadmill running). Nevertheless, our present results demonstrate a key role of increased vagal tone leading to sinus bradycardia in the swimming-trained rat model.

### Exercise training is associated with improved cardiac output and arrhythmia propensity

Intensive exercise requires increased cardiac output to satisfy the enhanced metabolic demand of the body. This could be associated with remodeling of several components of Ca^2+^ homeostasis^27–29^. In line with this, our in vivo and in vitro results unequivocally show increased LV pressure in the trained group (Fig. 2) which was tightly associated with larger incidence of ventricular spontaneous beats during Langendorff perfusion (Fig. 3). ECG analysis revealed the ventricular origin of the extra beats in isolated hearts, suggesting delayed afterdepolarizations mediated by Ca^2+^-overload, as the underlying mechanism. In this case, the premature beats are governed by facilitated forward NCX activity as a result of larger SR Ca^2+^ content^20,30,31^. The shorter coupling intervals of the premature beats in trained animals may support this hypothesis, since a higher SR Ca^2+^ content due to improved Ca^2+^ sequestration may lead to earlier spontaneous events (Fig. 4). Accordingly, in field-stimulated Ca^2+^ transient measurements in isolated cells, a markedly larger spontaneous Ca^2+^ release activity was found in the trained group that could cause Ca^2+^-driven extra depolarizations mediated by the NCX (Fig. 5).

### Training induced complex remodeling of SR proteins could be responsible to larger Ca^2+^ content

Experiments with rapid application of caffeine revealed significantly larger Ca^2+^ content of the SR in trained animals (Fig. 6b). The actual content of the SR is determined by the dynamic balance between the uptake (via SR Ca-ATPase, SERCA) and release through the ryanodine receptors. Phospholamban (PLN), the regulatory protein of SERCA binds to SERCA in its unphosphorylated form reducing its activity. PLN exists in monomeric and pentameric forms. While it is suggested that the monomeric form inhibits SERCA^32^, the role of pentamers is still unclear. This model is derived from the observation that monomers are better inhibitors of PLN^33,34^, however, it was also found that a dynamic equilibrium exists between monomeric and pentameric forms. Furthermore, phosphorylation of SERCA or increased cytosolic Ca^2+^ level increased the proportion of the pentameric pool at the expense of monomeric pool^35^. Taken together, several independent reports claim that SERCA may interact with oligomeric forms of PLN, and the existence of oligomers appears to offer a functional advantage for the SERCA-PLN interaction^36–40^. In line with these findings, our results suggest that the increase of PLN pentameric form is associated with improved SERCA kinetics, providing faster decay of the Ca^2+^ transient, and larger Ca^2+^ content and possible Ca^2+^ overload of the sarcoplasmic reticulum (Fig. 6b–e) that may serve as an arrhythmogenic trigger source by spontaneous Ca^2+^ release. Furthermore, we found enhanced expression level of calsequestrin, an ubiquitous luminal Ca^2+^ binding protein^41^ (Fig. 8). It has been proposed that CASQ binds to ryanodine receptor via triadin or junctin, and when free [Ca^2+^] is low in the SR, ryanodine receptor open probability is reduced. When SR [Ca^2+^] increases, CASQ dissociates from the ryanodine receptor increasing its open probability^42^. It has been shown that CASQ intimately determines the magnitude and duration of the Ca^2+^ release from the SR^43^ providing a local source of the releasable Ca^2+^ and it has an important role in ryanodine receptor gating. It was further demonstrated that CASQ modulated ryanodine receptor function via the interaction of two further intraluminal proteins, triadin and junctin^44–46^. Our results indicate that the increased SR Ca^2+^ content is maintained by the increased expression level of CASQ, providing the molecular basis for the improved cardiac output via enhancing the storage capacity and the amount of releasable Ca^2+^ from the SR.

### Identical repolarizing currents between trained and control groups

Crucial repolarizing currents, such as I_to_ and I_K1_, did not differ in cardiomyocytes isolated form trained and control animals (Fig. 7). In our rat model, exercise induced electrophysiological remodeling is confined to Ca^2+^ homeostasis and arrhythmias were promoted by increased triggering activity. It is important to note, however, that potassium channel remodeling might occur in large mammals and may critically alter transmural dispersion of potassium currents, providing an enhanced arrhythmogenic substrate. Considering that several potassium channels show Ca^2+^ dependence to some extent^47^, the indirect influence of Ca^2+^ homeostasis alterations on repolarization may also play an important role, and in turn may explain the increased long-term ECG QT variability in the trained group (Table 2).

## Conclusion

Our results lead us to conclude that sudden cardiac death associated with training-induced remodeling could possibly arise as the disadvantageous consequence of Ca^2+^ homeostasis adaptation in the athlete’s heart. The increased Ca^2+^ content of the SR provides larger available Ca^2+^ upon its release, which is an adaptive response to meet the enhanced cardiac output demand during exercise. However, the enhanced Ca^2+^ load of the SR in trained hearts may also serve as a potential arrhythmia trigger source inducing spontaneous Ca^2+^ release events. The spontaneous Ca^2+^ release could be amplified the presence of increased sympathetic tone or electrolyte disturbances during training, exposing cardiomyocytes to extra Ca^2+^ load. The enhanced source of arrhythmogenic triggers, coupled with possible impaired repolarization reserve in human may lead to the development of life threatening arrhythmias such as ‘*torsades de pointes*’ tachyarrhythmia or ventricular fibrillation in top athletes.

### Limitations

(i) Our rat model did not show alterations of the repolarization process, transmural dispersion and therefore changes in the arrhythmia substrate could not be observed. This observation may derive from the species-dependent characteristics of repolarization. Since the rat action potential repolarization relies on I_to_ and I_K1_, I_Kur_ (Kv1.5), the action potential prolonging effect of a possible I_Kr_ and I_Ks_ downregulation could not be detected, and should be studied experimentally in other species like rabbit, guinea-pig or dog. (ii) In addition, the Ca^2+^ removal from the intracellular space in rats depends more on the SERCA activity compared to larger species, including human.

## Abbreviations

AWd: Anterior wall in diastole
AWs: Anterior wall in systole
AWT: Anterior wall thickness
CASQ: Calsequestrin
EDD: END-diastolic diameter
ESD: End-systolic diameter
FS: Fractional shortening
GAPDH: Glyceraldehyde-3-phospate dehydrogenase
HR: Heart rate
I_CaL_: L-type calcium current
IgG: Immunoglobulin G
I_K1_: Inward rectifier potassium current
I_Kr_: Rapid component of the delayed rectifier potassium current
I_Ks_: Slow component of the delayed rectifier potassium current
I_to_: Transient outward potassium current
LTCC: L-type calcium channel
LV: Left ventricular
LVAWTd: Left ventricular anterior wall thickness at diastole
LVAWTs: Left ventricular anterior wall thickness at systole
LVDP: Left ventricular developed pressure
LVEDD: Left ventricular end-diastolic diameter
LVEDP: Left ventricular end-diastolic pressure
LVESD: Left ventricular end-systolic diameter
LVESP: Left ventricular end-systolic pressure
LVP: Left ventricular pressure
LVPWTd: Left ventricular posterior wall thickness at diastole
LVPWTs: Left ventricular posterior wall thickness at systole
NCX: Sodium/calcium exchanger (Na^+^/Ca^2^^+^ exchanger)
PLN: Phospholamban
pPKAC: Phospho-protein kinase A C
pPLN: Phospho-phospholamban
PWd: Posterior wall in diastole
PWs: Posterior wall in systole
PWT: Posterior wall thickness
Ryr: Ryanodine receptor
SERCA2a: Sarcoplasmic reticulum Ca^2^^+^-ATPase
TL: Tibial length
