# Author Correction: Increased Ca^2+^ content of the sarcoplasmic reticulum provides arrhythmogenic trigger source in swimming-induced rat athlete’s heart model

**DOI:** 10.1038/s41598-021-91695-1

**Published:** 2021-06-04

**Authors:** Péter Gazdag, Kinga Oravecz, Károly Acsai, Vivien Demeter‑Haludka, Balázs Ördög, Jozefina Szlovák, Zsófia Kohajda, Alexandra Polyák, Bálint András Barta, Attila Oláh, Tamás Radovits, Béla Merkely, Julius Gy. Papp, István Baczkó, András Varró, Norbert Nagy, János Prorok

**Affiliations:** 1 Department of Pharmacology and Pharmacotherapy, Faculty of Medicine, University of Szeged, Dóm tér 12, P.O. Box 427, Szeged, 6720 Hungary; 2 MTA‑SZTE Research Group of Cardiovascular Pharmacology, Hungarian Academy of Sciences, Szeged, Hungary; 3 2Nd Department of Internal Medicine and Cardiology Centre, Faculty of Medicine, University of Szeged, Szeged, Hungary; 4 Experimental Research Laboratory, Heart and Vascular Center, Semmelweis University, Budapest, Hungary; 5 Department of Pharmacology and Pharmacotherapy, Interdisciplinary Excellence Centre, University of Szeged, Szeged, Hungary

Correction to: *Scientific Reports* 10.1038/s41598-020-76496-2, published online 11 November 2020

This Article contains errors in the Results section under subheading ‘The I_Ca,L_, SR Ca^2+^ content and Ca^2+^ transient measurements’.

“We analyzed the integral of caffeine-induced NCX currents as an indicator of the SR Ca^2+^ content and found that SR Ca^2+^ content was significantly increased in the trained group compared to controls (− 1.84 ± 0.4 (pA*s)/pF vs − 1.25 ± 0.5 (pA*s)/pF n = 8/5 and 8/4, respectively, *p* < 0.05; Fig. 6b).”

should read:

“We analyzed the integral of caffeine-induced NCX currents as an indicator of the SR Ca^2+^ content and found that SR Ca^2+^ content was significantly increased in the trained group compared to controls (− 1.84 ± 0.4 (pA*s)/pF vs − 1.25 ± 0.5 (pA*s)/pF n = 8/5 and 8/5, respectively, *p* < 0.05; Fig. 6b).”

Secondly, this Article contains an error in Figure 6 where the experimental numbers of the control columns are incorrect (8/4) in panel (b). The correct Figure 6 appears below as Figure 1.

**Figure 1:**
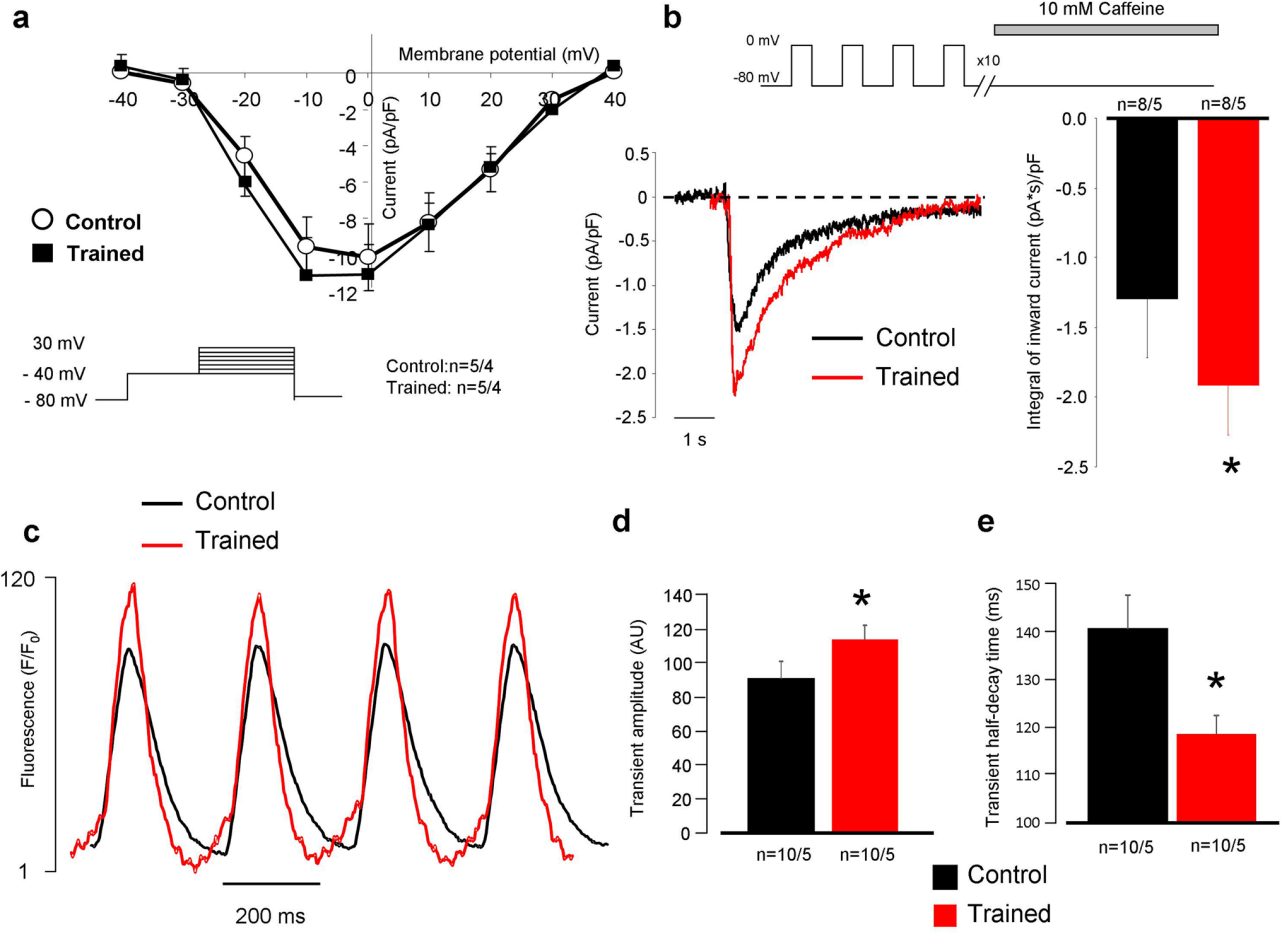
Assessment of Ca^2+^ handling on isolated cells. Panel (**a**) shows identical current–voltage relationship of L-type Ca^2+^ current between groups. Panel (**b**) illustrates significantly larger inward current as a response of 10 mM caffeine application. Panel (**c**,**d**) reports larger Ca^2+^ transient amplitude in the case of trained rats (red trace) compared to control (black trace). Panel (**e**) indicates faster transient relaxation kinetics in the case of trained animals.

